# A room-temperature sodium rechargeable battery using an SO_2_-based nonflammable inorganic liquid catholyte

**DOI:** 10.1038/srep12827

**Published:** 2015-08-05

**Authors:** Goojin Jeong, Hansu Kim, Hyo Sug Lee, Young-Kyu Han, Jong Hwan Park, Jae Hwan Jeon, Juhye Song, Keonjoon Lee, Taeeun Yim, Ki Jae Kim, Hyukjae Lee, Young-Jun Kim, Hun-Joon Sohn

**Affiliations:** 1 Advanced Batteries Research Center, Korea Electronics Technology Institute, Seongnam, 463-816, Korea; 2 Department of Energy Engineering, Hanyang University, Seoul, 133-791, Korea; 3 CAE Group, Samsung Advanced Institute of Technology, Yongin, 446-712, Korea; 4 Department of Energy and Materials Engineering, Dongguk University-Seoul, Seoul, 100-715, Korea; 5 School of Materials Science and Engineering, Andong National University, Andong, 760-745, Korea; 6 Department of Materials Science and Engineering, Seoul National University, Seoul, 151-744, Korea

## Abstract

Sodium rechargeable batteries can be excellent alternatives to replace lithium rechargeable ones because of the high abundance and low cost of sodium; however, there is a need to further improve the battery performance, cost-effectiveness, and safety for practical use. Here we demonstrate a new type of room-temperature and high-energy density sodium rechargeable battery using an SO_2_-based inorganic molten complex catholyte, which showed a discharge capacity of 153 mAh g^**−**1^ based on the mass of catholyte and carbon electrode with an operating voltage of 3 V, good rate capability and excellent cycle performance over 300 cycles. In particular, non-flammability and intrinsic self-regeneration mechanism of the inorganic liquid electrolyte presented here can accelerate the realization of commercialized Na rechargeable battery system with outstanding reliability. Given that high performance and unique properties of Na–SO_2_ rechargeable battery, it can be another promising candidate for next generation energy storage system.

In order to address recent concerns on the limited resources of lithium and the localized reserves, Na rechargeable batteries have gained much attention as alternative power sources to replace Li rechargeable ones. Up to date, several types of Na rechargeable batteries have been investigated, such as, high-temperature Na–S (NAS) and Na–NiCl_2_ (ZEBRA) batteries, room-temperature Na-ion and Na–O_2_ batteries, and each system has the pros and cons; more detailed materials and technology for the Na rechargeable batteries are well discussed in other recent works^1^,^2^,^3^,^4^,^5^,^6^,^7^,^8^. Herein, we demonstrate a new type of Na rechargeable battery using an SO_2_-based inorganic molten complex as both (i) a Na^+^-conducting medium and (ii) cathode material, i.e. catholyte, suggesting as an alternative room-temperature and high-energy Na rechargeable battery. In the history of batteries, SO_2_ is not a strange material for Li batteries. Primary Li–SO_2_ batteries in which liquefied SO_2_ serves as the active cathode material have been commercialized for military and industrial applications^9^,^10^, and ongoing interest for further development is still found^11^. About 30 years ago, there were also intensive studies on Li–SO_2_ rechargeable batteries based on a LiAlCl_4_⋅*x*SO_2_ inorganic molten complex catholyte, which shows completely different reaction chemistry from the primary SO_2_ battery^8^,^12^,^13^,^14^,^15^,^16^. Rechargeable Li–SO_2_ battery showed a discharge capacity of ~1000 mAh g^−1^ based on the carbon electrode (theoretical catholyte capacity of 144 mAh g^−1^ for LiAlCl_4_⋅6SO_2_) with an operating voltage of 3.2 V, and Duracell demonstrated the performance of prototype C-size Li–SO_2_ rechargeable batteries^12^. One of the key advantages of Li–SO_2_ rechargeable battery is the use of a highly conductive electrolyte (~0.1 S cm^−1^ at room temperature)^17^, which is almost same to typical ionic conductivity of aqueous electrolytes. This excellent conductivity of Li^+^ ensures a high electrochemical reversibility and rate capability of rechargeable Li–SO_2_ battery system. Moreover, the inorganic electrolyte employed in Li–SO_2_ battery is non-flammable^18^, offering additional attractive feature to Li–SO_2_ battery over other flammable organic electrolyte-based Li batteries. The Li–SO_2_ rechargeable battery, however, could not succeed in its commercialization for consumer application, mainly because the use of LiAlCl_4_⋅6SO_2_ resulted into high internal cell pressure, which raised the safety concerns about cell venting even under moderate cycling condition^12^,^19^; specifically, the equilibrium vapor pressure of SO_2_ for LiAlCl_4_⋅6SO_2_ is about 2 bar at 20 °C (~7 bar at 60 °C) and, moreover, the LiAlCl_4_⋅6SO_2_ catholyte releases SO_2_ gas when the cell discharges in accordance with the reaction chemistry^12^,^13^,^17^. Otherwise, LiAlCl_4_⋅3SO_2_ (not 6SO_2_) shows relatively low equilibrium vapor pressure of ~1 bar at 20 °C (~2 bar at 60 °C) and involves no change in the cell internal pressure during discharge, which could be a better composition having a safety advantage^13^,^17^. It, however, crystallizes when cooled to about –10 °C and remains a solid even after heated up to 25 °C, because a solid phase of LiAlCl_4_⋅3SO_2_ is more stable for than its liquid phase at ambient temperature^17^,^20^.

NaAlCl_4_⋅*x*SO_2_, a homologue of LiAlCl_4_⋅*x*SO_2_, was introduced by Kühnl *et al.* in the 1970s^21^,^22^ as a highly conductive Na^+^ electrolyte (~0.1 S cm^−1^); however, this electrolyte has never been explored for Na rechargeable batteries although it has distinguishable properties from LiAlCl_4_⋅*x*SO_2_. NaAlCl_4_⋅2SO_2_, known as a stable composition under ambient conditions^22^, exhibits the equilibrium vapor pressure of ~1 bar and remains as a liquid phase up to –40 °C without freezing, thus alleviating our safety concerns regarding cell venting that was a critical issue in the past Li–SO_2_ battery^17^,^21^,^22^. These physicochemical properties of NaAlCl_4_⋅2SO_2_ motivated us to study and develop a Na–SO_2_ battery system, particularly for low-cost stationary power storage applications. Here we report a Na–SO_2_ rechargeable battery system using NaAlCl_4_⋅2SO_2_ electrolyte. We found that the optimized carbon cathode enables a reversible reaction of the catholyte with high capacity, good rate capability, a long life-span over 300 cycles, and an estimated theoretical energy density of 407 Wh kg^−1^ (based on the discharged product including carbon cathode). This value is comparable with those of other high-energy Na rechargeable batteries. Moreover, non-flammability, low vapor pressure, and unique self-regeneration mechanism of the inorganic electrolyte presented here would be noteworthy merits of Na–SO_2_ system over other Na rechargeable battery systems.

## Results

**Figure 1:**
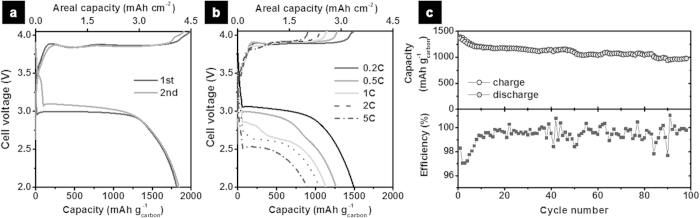
Electrochemical performance of Na–SO_2_ cells. (**a**) The first and second galvanostatic voltage profiles of a Na–SO_2_ cell at 0.1C (=150 mA g^−1^ and 0.34 mA cm^−2^). The cutoff voltage for charge is 4.05 V. (**b**) Discharge rate capability of a Na–SO_2_ cell. The charging rate is fixed at 0.2C. The capacity retention during the rate capability test was displayed in Supplementary Figure S1. (**c**) Cycle performance of a Na–SO_2_ cell at a rate of 0.5C discharge and 0.2C charge for 100 cycles.

In this work, we constructed a 2032 coin-type Na–SO_2_ cell using a Na-metal anode and a porous carbon cathode with NaAlCl_4_⋅2SO_2_ as a catholyte. The carbon cathode was prepared by roll-pressing of ketjenblack/polytetrafluoroethylene paste on a Ni-mesh, and NaAlCl_4_⋅2SO_2_ was synthesized by blowing SO_2_ gas through a mixture of NaCl and AlCl_3_ powders. Details of materials and experimental methods are described in Methods. Figure 1a shows the first and second voltage profiles of the Na–SO_2_ cell that delivers a discharge capacity of ~1800 mAh g^−1^ based on the carbon cathode at a rate of 0.1C (=150 mA g^−1^ or 0.34 mA cm^−2^). This corresponds to an areal capacity of 4.1 mAh cm^−2^, which is comparable to typical values of commercial Li-ion batteries (3–5 mAh cm^−2^) and much higher than those of reported Li–O_2_ and Na–O_2_ batteries^6^,^23^,^24^,^25^,^26^. The Na–SO_2_ cell also showed an encouraging rate capability exhibited in Fig. 1b, where a high capacity of 897 mAh g^−1^ is observed even at a significantly high current density of 5C (7500 mA g^−1^ or 17 mA cm^−2^). Given that the rate capability is one of the most challenging issues in NAS, ZEBRA, and Na–O_2_ batteries^1^,^23^,^24^, the excellent power capability could give the Na–SO_2_ battery a critical edge over other Na rechargeable batteries previously reported. It should be also noted that the operating voltage of the Na–SO_2_ cell was ~3.0 V at 0.1 C. It is higher than those of NAS (2.0 V), ZEBRA (2.58 V), and Na–O_2_ (2.2–2.5 V), and also comparable to those of most Na-ion battery cathodes^1^,^2^,^3^,^4^,^5^,^6^,^7^,^26^. However, the voltage gap between discharge and charge was evident in the Na–SO_2_ system, and the low round-trip energy efficiency (~80%) needs to be further ameliorated. The Na–SO_2_ cell showed relatively good capacity retention during cycling, i.e. 75% of the initial capacity after 100 cycles (Fig. 1c), even under full depth-of-discharge condition, accompanied by high columbic efficiencies during cycling (average of ~99%).

**Figure 2:**
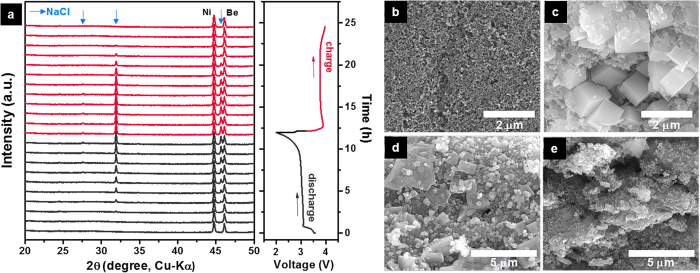
In situ XRD and SEM analysis on the Na–SO_2_ cell cathodes. (**a**) In situ XRD patterns of the carbon cathode in a Na–SO_2_ cell during the first cycle. The referred NaCl corresponds to JCPDS #780751, indicated by arrows. The SEM images of the carbon cathode in a Na–SO_2_ cell (**b**) before discharge (pristine), (**c**) after full discharge, (**d**) after 50% charge, and (**e**) after full charge.

It is generally accepted that the underlying reaction mechanism of the SO_2_-based catholyte is reversible changes in the oxidation state of sulfur in SO_2_ between +4 and +3 (ref. 13 and 14). The detailed reaction chemistry is, however, still unclear. To elucidate the electrochemical reaction responsible for the exceptional performance of the Na–SO_2_ cell, we performed various *in situ* and *ex situ* analyses. Figure 2a shows *in situ* X-ray diffraction (XRD) patterns of the carbon cathode during the first cycle. On discharge, new peaks corresponding to NaCl started to appear and their intensity increased as discharge continued. On subsequent charge, the NaCl peaks diminished gradually until they completely disappeared at the end of the charge. This reversible behavior of NaCl was further confirmed by scanning electron microscopy (SEM) and energy-dispersive X-ray spectroscopy (EDS) observations (Fig. 2b–e and Supplementary Figure S2). Well-defined cubic solid discharge products with a size of about 2 μm appeared after the discharge, and they vanished gradually during the successive charge. The NaCl crystals formed during discharge are regarded as the products of the electrochemical reduction of SO_2_ to SO_2_.^−^ radical anions which displace Cl^−^ from AlCl_4_^−^ to form NaCl. In a Li–SO_2_ battery, homologue of Na–SO_2_, LiCl and LiAlCl(SO_2_)_3_ have been considered as discharge products^13^: 3 moles of reduced SO_2_^.−^ anions react with 1 mole of AlCl_4_^−^ sequentially, thereby forming insoluble 3 moles of LiCl and 1 mole of LiAlCl(SO_2_)_3_, which precipitate at the carbon cathode. While LiCl was confirmed by XRD analysis, the SO_2_-substituted second form of the discharge product has not been identified clearly despite several efforts made in various experimental analyses^13^.

**Figure 3:**
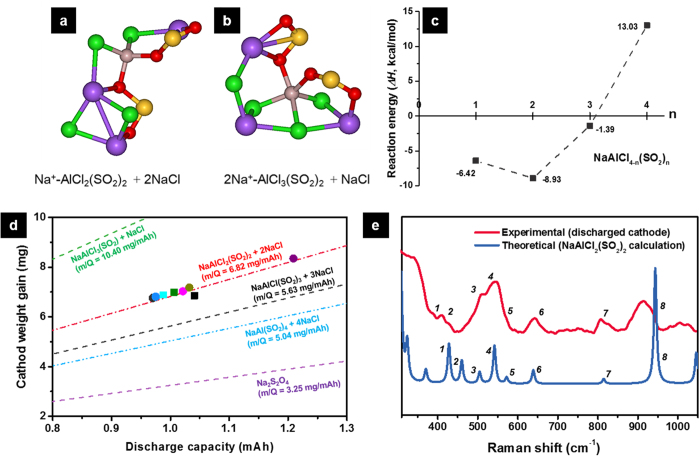
Theoretical calculation and SERS analysis on the Na–SO_2_ cell cathodes. The atomic structures of the discharge products with minimum energy during the AIMD simulation (grey: Al, green: Cl, red: O, yellow: S, purple: Na): (**a**) NaAlCl_2_(SO_2_)_2_ + 2NaCl, (**b**) Na_2_AlCl_3_(SO_2_)_2_ + NaCl. (**c**) The SO_2_^.−^ to Cl^−^ substitution reaction energies of NaAlCl_4_ from static first-principles calculations. (**d**) Relationship between cathode weight gain and discharge capacity in Na–SO_2_ cells. Several hypotheses depending on the numbers of SO_2_ reduced at the cathode were considered, including also Na_2_S_2_O_4_ formation. The theoretical mass-to-charge relationships were displayed as lines and the experimental measurements were plotted by scatters. (**e**) The observed SERS spectra of the discharged Na–SO_2_ cell cathode and comparison with the calculated Raman frequency. The numbered Raman frequencies with the vibrational assignment are given in Supplementary Table S1.

In the Na–SO_2_ cell, we also detected another type of product composed of Na, Al, Cl, S, and O elements at the carbon surface by using SEM-EDS (Supplementary Figure S2). To clarify the reaction pathway of the reduced SO_2_^.−^ anions and the chemical structure of the resulting discharge products in a Na–SO_2_ system, we performed an ab initio molecular dynamics (AIMD) simulation, combined with an experimental analysis of surface-enhanced Raman spectroscopy (SERS). As shown in the simulation snapshots of the reaction products that have minimum energy during discharge (Fig. 3a,b), the most stable structure of the discharge products is quadra-coordinated Al species bonded by an oxygen atom to SO_2_, i.e., NaAlCl_2_(SO_2_)_2_ with NaCl, while penta-coordinated Na_2_AlCl_3_(SO_2_)_2_ was occasionally observed during AIMD simulation and is considered as a minor discharge product. Consequently, the AIMD simulation sheds light onto how SO_2_^.−^ radical anions stabilize themselves by displacing chlorine anions from tetrachloroaluminates to form NaCl and SO_2_-complexes. Regarding to the possible mole number of SO_2_^.−^ displacing Cl^−^ from 1 mole of AlCl_4_^−^ during discharge, static first-principles calculations of the substitution reactions (Fig. 3c) support the above AIMD result that the substitution of 2 SO_2_ into AlCl_4_^−^, i.e., the formation of NaAlCl_2_(SO_2_)_2_ is the most feasible reaction. This stoichiometric behavior was experimentally confirmed by investigating the mass-to-charge ratio (m/Q) at the cathode after discharge, where the m/Q is defined as weight gain at the cathode per discharge capacity. The experimental m/Q value from our many repeated measurements was 6.89 ± 0.14 mg mAh^−1^ which is quite close to the value expected for the exclusive formation of NaAlCl_2_(SO_2_)_2_ and 2NaCl; the formation of NaAlCl_2_(SO_2_)_2_ and 2NaCl would consume 2 SO_2_ with 2 e^−^ per NaAlCl_4_ and result in a 6.82 mg mAh^−1^ cathode weight gain, as indicated by the slope of the red line in Fig. 3d.

To identify the SO_2_-substituted discharge product, we also carried out ex situ SERS measurement to probe the chemical structure of the discharge product and compared the calculated Raman spectra based on the aforementioned reaction mechanism, as presented in Fig. 3e. The observed Raman peaks in the spectral range between 400 and 600 cm^−1^, and at around 620, 800 and 920 cm^−1^ (the corresponding vibration modes are described in Supplementary Table S1) are relatively well matched with the calculated ones corresponding to NaAlCl_2_(SO_2_)_2_, suggesting that NaAlCl_2_(SO_2_)_2_ is the most plausible second discharge product formed at the cathode. Putting all the above results together, the full cell reaction scheme of the Na–SO_2_ rechargeable battery is proposed as follows:


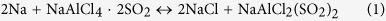


Based on the above reaction, the theoretical capacity of NaAlCl_4_⋅2SO_2_ is 168 mAh g^−1^ (or 147 mAh g^−1^ based on the discharge products). Nonetheless, since the reaction of the electroactive material is highly dependent on the physicochemical properties of the carbon cathode such as its surface area and pore structure, we estimated the theoretical energy density of a Na–SO_2_ battery based on the mass of discharge products including carbon cathode. The evaluated energy density is 407 Wh kg^−1^ (for details, see Supplementary Table S2), which is comparable to other high-energy Na rechargeable batteries^6^,^7^.

**Figure 4:**
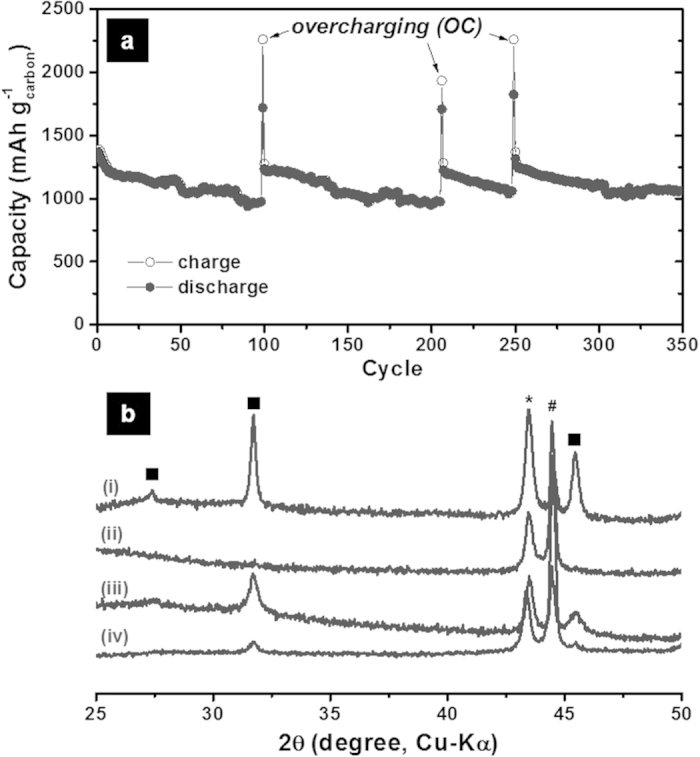
Long-term cycle performance of Na–SO_2_ cells with overcharging processes. (**a**) The cycle performance of a Na–SO_2_ cell for 350 cycles including overcharging processes. Overcharging was forced at the 99th, 206th and 249th cycles for a given time. The corresponding voltage profiles before and after the 99th overcharging are given in Supplementary Figure S5. (**b**) Ex situ XRD patterns of the carbon cathode in Na–SO_2_ cells before and after overcharging: (i) after the first discharge, (ii) after the first charge, (iii) after the 50th charge, and (iv) after the 51st overcharge (■ NaCl, *SUS holder, # Ni mesh).

The capacity fading of the Na–SO_2_ cell shown in Fig. 1c is mainly attributed to residual insulating discharge products that passivate carbon surface and/or block the pore entrance in the electrode, thereby reducing reaction site and increasing the impedance of the carbon cathode. We observed that NaCl did not disappear completely in the carbon cathode during repeated cycling, so that the accumulated discharge products increased the impedance of the cathode (Supplementary Figure S3 and S4). It is interesting that a tetrachloroaluminate:SO_2_ complex has an intrinsic self-regeneration mechanism^12^,^27^ which can be utilized to remove residual NaCl from the carbon cathode. When a Na–SO_2_ cell was overcharged to above 4.05 V, the recombination reaction took place as like a Li–SO_2_. According to the proposed overcharging mechanism for a Li–SO_2_ system^12^,^27^, the oxidation of AlCl_4_^−^ produces Cl_2_ and AlCl_3_ during overcharge. The highly soluble Cl_2_ gas dissolves into the electrolyte and reacts with the Na-metal anode to form NaCl, which further reacts with AlCl_3_ to regenerate NaAlCl_4_. The produced AlCl_3_ can also react with residual NaCl at the cathode to regenerate NaAlCl_4_. These recombination reactions during overcharge can facilitate the reactivation of the surface and the pore structure of the carbon cathode, thereby restoring capacity of the Na–SO_2_ cell. Figure 4a shows the cycle performance before and after overcharging (See also Supplementary Figure S5 for the corresponding voltage profiles). At the 98th cycle under the normal charge/discharge condition, the capacity was below 1000 mAh g^−1^. Surprisingly, it jumped up about 1250 mAh g^−1^ after overcharging up to 4.3 V at the 99th cycle and then showed stable cycle performance during the subsequent 100 cycles (~80% of capacity retention for the subsequent 100 cycles). The cell was further cycled up to 350 cycles with another two overcharging processes. After each overcharging process, the capacity came back its initial value and finally retained 80% of the initial capacity (1000 mAh g^−1^) at the 350th cycle, which exhibits the remarkable long-term cycle performance of Na–SO_2_ cell. In XRD analysis of the cathode, NaCl peaks were observed after the 50th charge due to the accumulation of NaCl on carbon over repeated cycles. However, these NaCl peaks receded dramatically after the overcharge (Fig. 4b), supporting a cathode recuperation by the above-stated recombination reactions. SEM observations also gave a solid proof for this reaction (Supplementary Figure S6).

**Figure 5:**
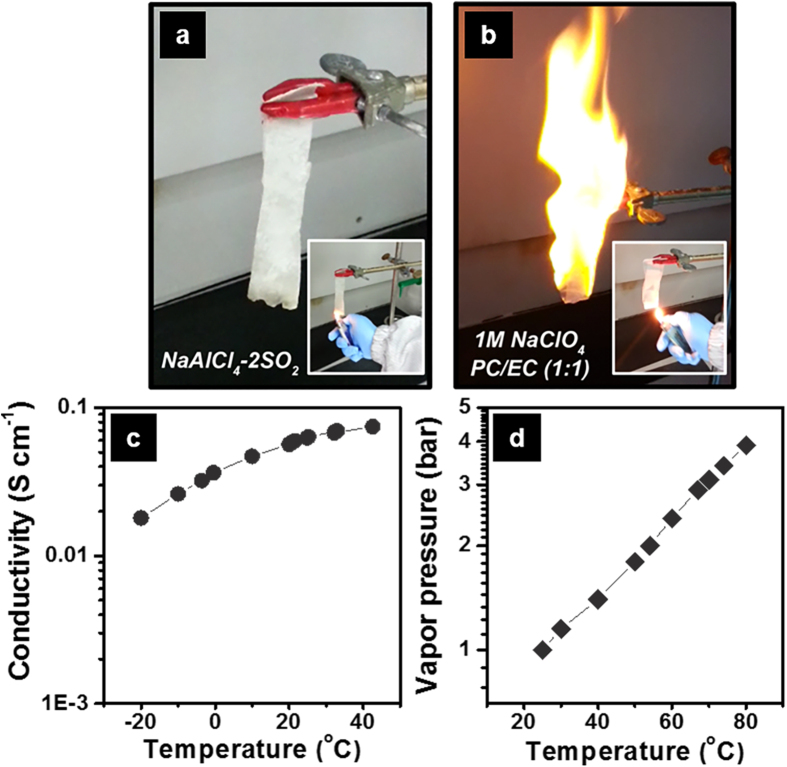
Physical and electrical properties of NaAlCl_4_.2SO_2_ inorganic electrolyte. Photo-snapshots of flammability-tests for (**a**) NaAlCl_4_⋅2SO_2_ inorganic electrolyte and (**b**) 1 M NaClO_4_ in the mixture of propylene carbonate (PC) and ethylene carbonate (EC) as one of organic-based electrolytes. The electrolyte-soaked tissue was forced to be contact with an open flame. (**c**) Conductivity of NaAlCl_4_⋅2SO_2_ electrolyte at various temperatures. (**d**) Vapor pressure of NaAlCl_4_⋅2SO_2_ electrolyte at various temperatures.

Finally, it should be emphasized that the reliability of a Na–SO_2_ battery is a major attractive feature over other Na rechargeable batteries. First of all, a Na–SO_2_ battery is working at ambient temperature. Considering NAS and ZEBRA batteries need the complicated implementation to ensure durability and safety due to high temperature (~300 °C) operation^1^, there would be no extra high capital cost for the system construction, and also no safety concern about seriously-reactive molten Na anode for the Na–SO_2_ battery. In comparison with other room-temperature Na-ion or Na–O_2_ batteries in which flammable organic solvents are normally used, the SO_2_-based inorganic electrolyte for the Na–SO_2_ battery is nonflammable, even in direct contact with an open flame (Fig. 5a,b). This self-extinguishing property of the electrolyte could significantly relieve the safety concerns about cell ignition or explosion of a Na–SO_2_ battery. Another important feature of a Na–SO_2_ battery stems from the still high Na^+^ conductivity of NaAlCl_4_⋅2SO_2_ at low temperatures (Fig. 5c). Owing to the excellent conductivity, a Na–SO_2_ cell could deliver a capacity of 1270 mAh g^−1^ and 830 mAh g^−1^ at 0 °C and –20 °C, respectively (Supplementary Figure S7). This reasonable low temperature performance with the NaAlCl_4_⋅2SO_2_ obviates a need for further increase of SO_2_ in case of a Na–SO_2_ system unlike a Li–SO_2_, and therefore, another safety concern regarding cell venting that was a critical issue in the past Li–SO_2_ battery could be relieved in a Na–SO_2_ battery. Figure 5d exhibits the vapor pressure of a NaAlCl_4_⋅2SO_2_ electrolyte at various temperatures. The vapor pressure at room-temperature is <1 bar (also, ~2 bar at 60 °C) and significantly lower than those of LiAlCl_4_⋅6SO_2_ and pure liquid SO_2_^17^.

## Discussion

We presented here a 3-V-class Na–SO_2_ battery delivering high discharge capacity, excellent rate capability, and long cycle-life. We firmly believe that these key battery performances of the Na–SO_2_ system are much more promising compared with other Na rechargeable batteries ever reported. We also demonstrated that the cell chemistry is based on the highly reversible redox reaction of SO_2_ with tetrachloroaluminate and the use of the NaAlCl_4_⋅2SO_2_ inorganic electrolyte enables highly reliable Na–SO_2_ system in terms of long cycle life as well as safety. For practical application, however, there still remain several problems to be resolved: the large voltage-hysteresis during discharge and charge, instability of Na-metal anodes or search for alternative anode materials, etc. Further studies for fundamental understanding of a Na–SO_2_ battery, such as clarifying a detailed reaction pathway during charge of a Na–SO_2_ battery, should be also needed. However, the recent advanced battery-technologies regarding materials, electrodes, cell engineering, and also state-of-the-art analytical methods, which have remarkably developed since the advent of lithium-ion batteries, could accelerate our research and development for an advanced Na–SO_2_ battery, as already observed in the recent research activities for reviving Li(or Na)–O_2_ and Li(or Na)–S systems^23^,^24^,^25^,^26^. Considering the many favorable features and promises discussed in this report, the Na–SO_2_ battery can be a viable system for next cost-effective energy storage system. Further, the SO_2_-based inorganic electrolyte can be widely applied to battery systems adopting other metallic anodes like Ca, K, Al, and Mg, which paves the way for the development of various non-lithium metal-based battery systems.

## Methods

### Synthesis of NaAlCl_4_·*x*SO_2_ electrolyte

NaCl (>99.9%, Alfa Aesar) was vacuum-dried at 120 °C for 24 hours before using, while anhydrous AlCl_3_, (99.999%, Alfa Aesar) was used without any purification. The electrolyte was prepared by blowing SO_2_ gas (anhydrous, Fluka) through a mixture of NaCl and AlCl_3_ in a glass/Teflon vessel. The molar ratio of NaCl to AlCl_3_ was 1.1 to avoid the presence of free AlCl_3_, which is known to be corrosive to alkali metals. As soon as SO_2_ gas contacted with the mixture, it became liquid of transparent light ocher color. The SO_2_ gas was blown until the desired SO_2_ concentration, which was determined by weighing the electrolyte vessel, was reached. The reaction-completed electrolyte vessel was transferred back into the Ar-filled glove box, and placed in a glass-bottle containing small pieces of Na metal to remove the possible AlCl_3_ residue or H_2_O.

### Electrode/cell fabrication

A carbon cathode was made of Ketjenblack (KB, EC-600JD) with 10% polytetrafluoroethylene (PTFE) binder. The paste was roll-pressed on Ni mesh and vacuum-dried at 200 °C for 1 hour. The loading level was 2.0–2.5 mg cm^−2^ and the electrode density was 0.2 g cm^−3^. A Na metal sheet as an anode was prepared by flattening a Na metal piece (Sigma-Aldrich) in an Ar-filled glove box. A glass microfiber filter of 190 μm thickness (GC50, Advantec) was used as a separator. A 2032 coin cell consisting the electrodes, separator, and NaAlCl_4_-2SO_2_ electrolyte was assembled in an Ar-filled glove box for discharge/charge tests. Beaker-type or swagelock-type cells were used for some occasions.

### Electrochemical test

The assembled cells were aged for 12 hours at room temperature and then electrochemically tested using a TOSCAT battery measurement system under the following protocols. The first and second cycles were operated galvanostatically at 0.1C (=150 mA g^−1^ or 0.34 mA cm^−2^) within the voltage window of 2.0–4.05 V. In the following cycles the current was set to be at 0.5C and 0.2C for discharge and charge, respectively. For rate capability test, the discharge rate was varied from 0.2C to 5C with a fixed charge rate at 0.2C. For an overcharging test, a Na–SO_2_ cell was charged up to 4.3 V and/or limited time. To investigate the impedance behavior of the carbon cathode, a 3-electrode electrochemical cell was constructed, where a Na metal reference electrode was positioned closely to the carbon cathode. Electrochemical impedance spectroscopic measurements conducted in the frequency range of 100 kHz to 10 mHz, with an amplitude of 5 mV at every end of charge and discharge step during cycling (VSP-300, BioLogic).

### Characterization

XRD (both *in situ* and *ex situ*) patterns were obtained using an Empyrean diffractometer (PANalytical) equipped with monochromated Cu Kα radiation (λ = 1.54056 Å). A lab-made swagelok-type *in situ* XRD cell was composed of KB-PTFE(10%) cathode, Na metal sheet anode, and glass fiber separator, with a beryllium (Be) disk on the cathode side for a X-ray window as well as a current collector. For ex situ analyses, a gas-tight sample holder filled with Ar and covered with a polyimide (Kapton) tape was used. After cell reacted up to certain level, the cathode was carefully disassembled from the cell and then rinsed with SOCl_2_ in an Ar-filled glove box to remove residual electrolyte since the SOCl_2_ is known to dissolve SO_2_ and NaAlCl_4_ well^28^. The morphology change of electrode after cycling was analyzed by SEM (JSM-7000F, JEOL). Weight gain at the cathode as a result of products formation was measured by weighing the carefully washed and dried cathode before and after discharge in an Ar-filled glove box. For the ex situ SERS measurement, we employed the gold(Au)-nanoparticles-anchored carbon black (Au@Vulcan XC-72) as the cathode, which was received from Nara Cell Tech Corp., Korea. The size and content of the Au nanoparticles were about 10–30 nm and 60 wt%, respectively, and the generation of SERS effect from the Au@C nanocomposite was confirmed before the measurement. The carefully washed and dried cathode was placed within a sealed sample holder where a quartz window was applied to the top cap. All procedures were undertaken in an Ar-filled glove box with O_2_ and H_2_O levels maintained at <1 ppm. SERS spectra were collected using a micro-Raman spectrometer (Bruker Senterra Grating 400) with a He–Ne laser at a wavelength of 532 nm. The power of the laser beam was less than 5 mW and the spectrum acquisition time was 10 s with 10 accumulations to avoid degradation to the standards or electrodes. The TEM image showing the size and population of Au-nanoparticles in the Au@C nanocomposite, and the discharge voltage profile of the Au@C cathode for the SERS measurement in Fig. 3e, are presented in Supplementary Figure S8.

### Computational Details

We conducted ab initio molecular dynamics (AIMD) simulations using the Vienna Ab initio Simulation Package^29^ (VASP) with the projector augmented-wave^30^ (PAW) approach for electrochemical reaction calculations. For the total energy calculation, the Perdew−Burke−Ernzerhof (PBE) generalized-gradient approximation (GGA) functional^31^ was used. On electrochemical reaction calculation, the electrons were added first into the initial structures composed of 2NaAlCl_4_ and 4SO_2_, and then Na atoms were added. We used Parrinello−Rahman dynamics for NPT ensemble and Minimal Γ-centered 1 × 1 × 1 k-point grid. Two or three snap shots, which had the minimum energy, were selected from AIMD simulations. Static first-principles calculations were calculated with the B3LYP functional and 6-31G(d) basis sets. The reaction energy and Raman frequency calculations were performed using the Gaussian09 program package.

## Additional Information

**How to cite this article**: Jeong, G. *et al.* A room-temperature sodium rechargeable battery using an SO_2_-based nonflammable inorganic liquid catholyte. *Sci. Rep.* **5**, 12827; doi: 10.1038/srep12827 (2015).

## Supplementary Material

Supplementary Information
