# Supplementary material for: A room-temperature sodium rechargeable battery using an SO2-based nonflammable inorganic liquid catholyte
Source: Sci Rep. 2015 Aug 5;5:12827. doi: 10.1038/srep12827 (PMC4525335; doi:10.1038/srep12827)

Supplementary Information for

A room-temperature sodium rechargeable battery using an SO_2-based nonflammable inorganic liquid catholyte

Goojin Jeong[1], Hansu Kim[2,*], Hyo Sug Lee[3], Young-Kyu Han[4], Jong Hwan Park[1,5], Jae Hwan Jeon[1,2], Juhye Song[1,2], Keonjoon Lee[4], Taeeun Yim[1], Ki Jae Kim[1], Hyukjae Lee[5], Young-Jun Kim[1,*] & Hun-Joon Sohn[6]

[1] Advanced Batteries Research Center, Korea Electronics Technology Institute, Seongnam, 463-816, Korea

[2] Department of Energy Engineering, Hanyang University, Seoul, 133-791, Korea

[3] CAE Group, Samsung Advanced Institute of Technology, Yongin, 446-712, Korea

[4] Department of Energy and Materials Engineering, Dongguk University-Seoul, Seoul, 100-715, Korea

[5] School of Materials Science and Engineering, Andong National University, Andong, 760-745, Korea

[6] Department of Materials Science and Engineering, Seoul National University, Seoul, 151-744, Korea

*Correspondence and requests for materials should be addressed to H.K. (khansu@hanyang.ac.kr) or Y.-J.K. (yjkim@keti.re.kr)

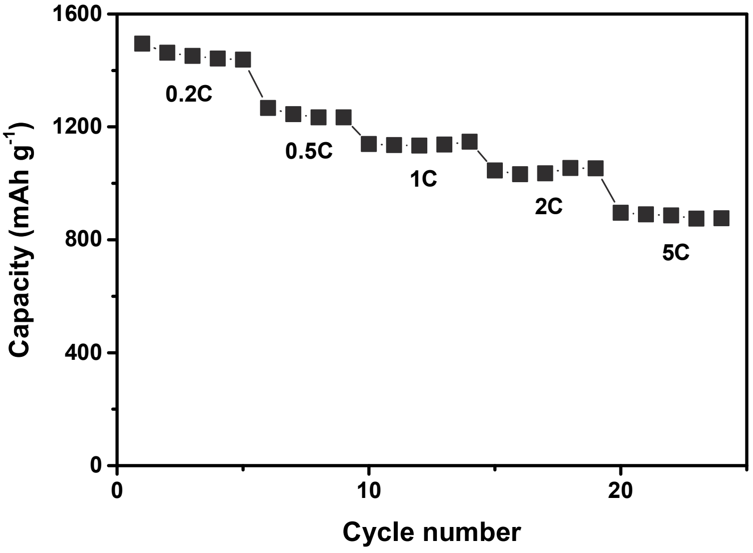

Figure S1. Capacity retention during the rate capability test shown in Figure 1b: Charging rate was fixed at 0.2C.

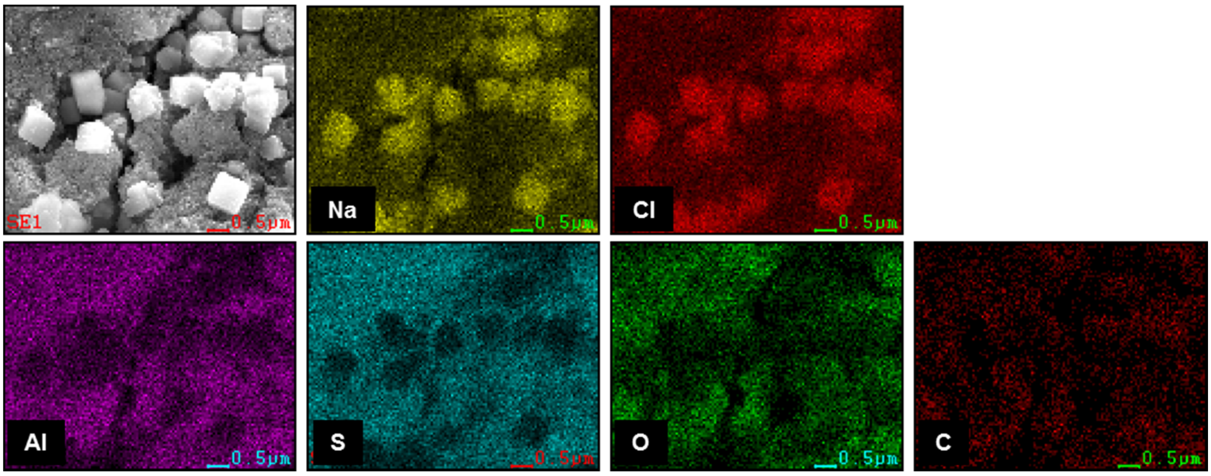

Figure S2. SEM-EDS mapping for the discharged carbon cathode in a Na–SO_2 cell: Na, Al, Cl, S, and O elements can be found on carbon surface around the cubic NaCl crystals.

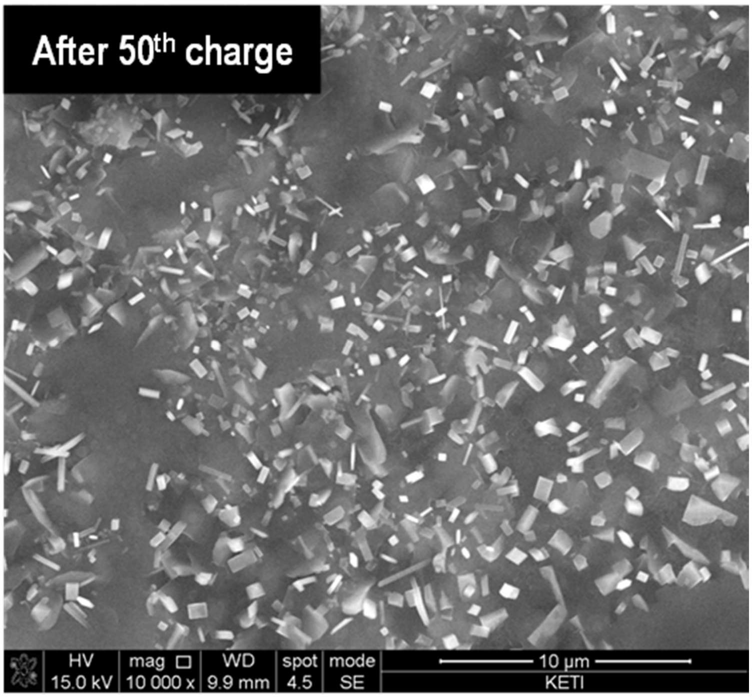

Figure S3. A SEM image of the carbon cathode of a Na–SO_2 cell after the 50th charge-step: The accumulated residual NaCl crystals could be observed even after the charge step.

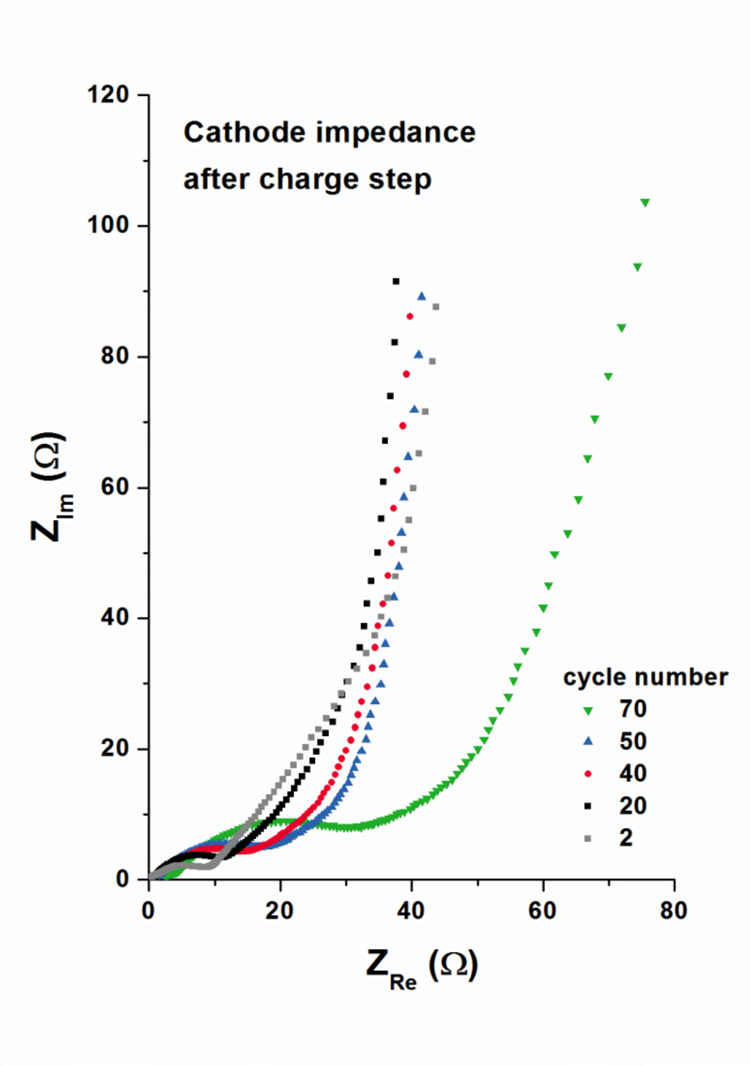

Figure S4. Nyquist plots of the carbon cathode in a Na–SO_2 cell after various cycles: The impedance measurement was carried out after charge step of each cycle in a three-electrode electrochemical cell.

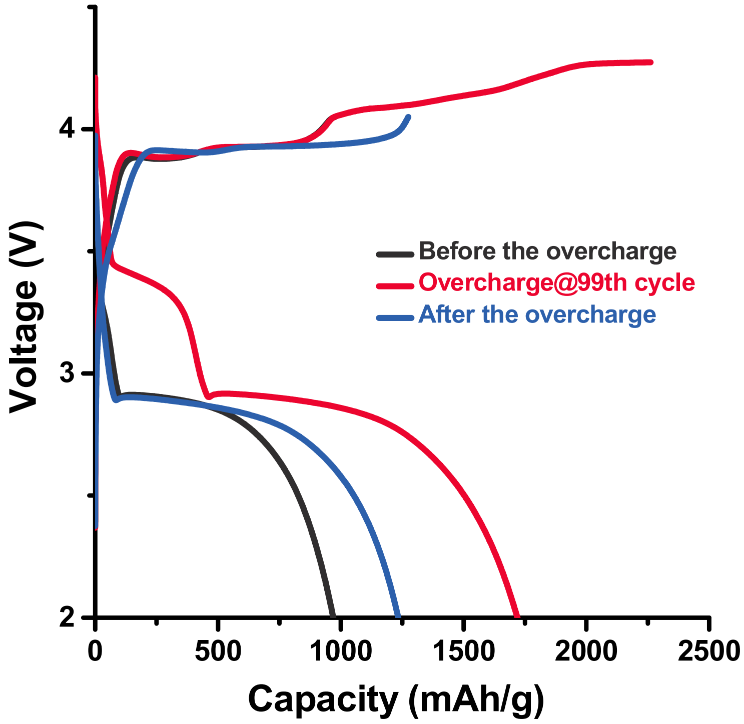

Figure S5. The voltage profiles of a Na–SO_2 cell before and after the 99th overcharging: just after the overcharging step, a voltage plateau at around 3.4 V was observed, which corresponds to a reduction of dissolved Cl_2 residue produced in the prior overcharging step.[12]

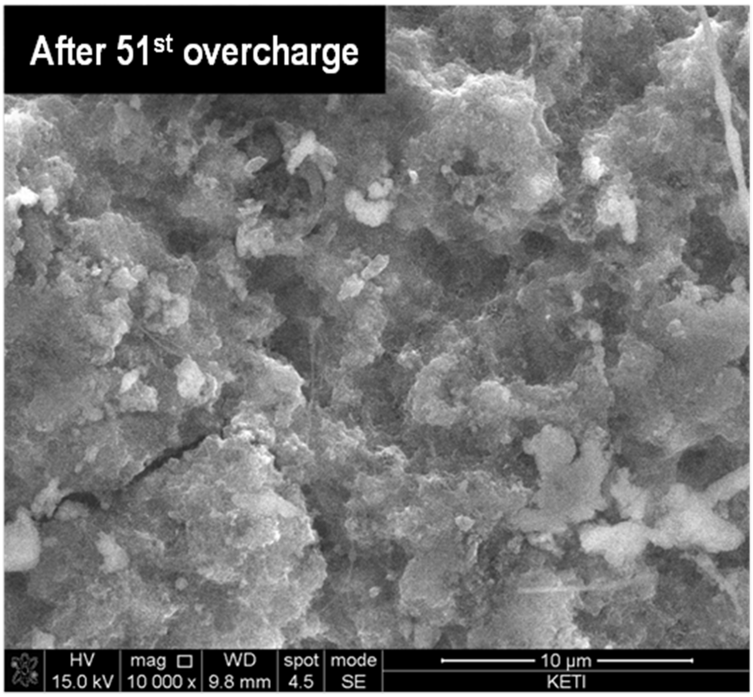

Figure S6. A SEM image of the carbon cathode after an overcharging step: Compared with the Figure S3, NaCl crystals are hardly found, indicating recuperation of the carbon cathode by the overcharging.

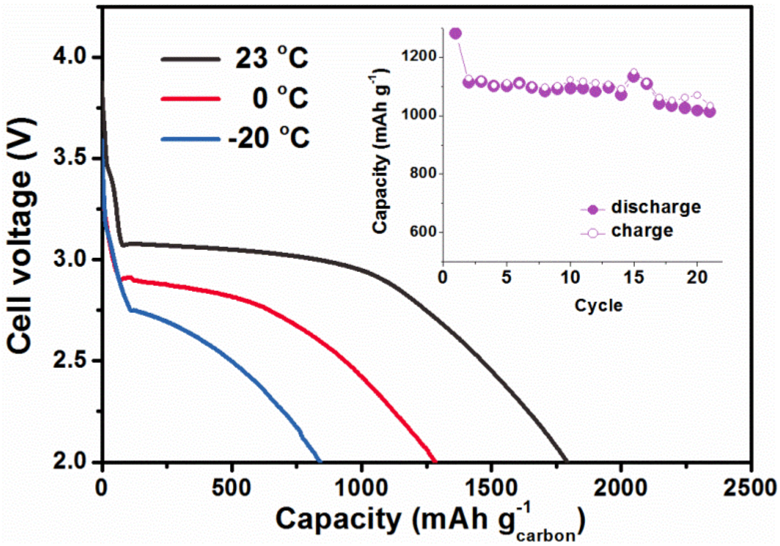

Figure S7. Discharge voltage profiles of a Na–SO_2 cell operated at various low temperatures. The inset shows the cycle performance at 0 ºC with a current density of 0.1C and a charging voltage of 4.2 V.

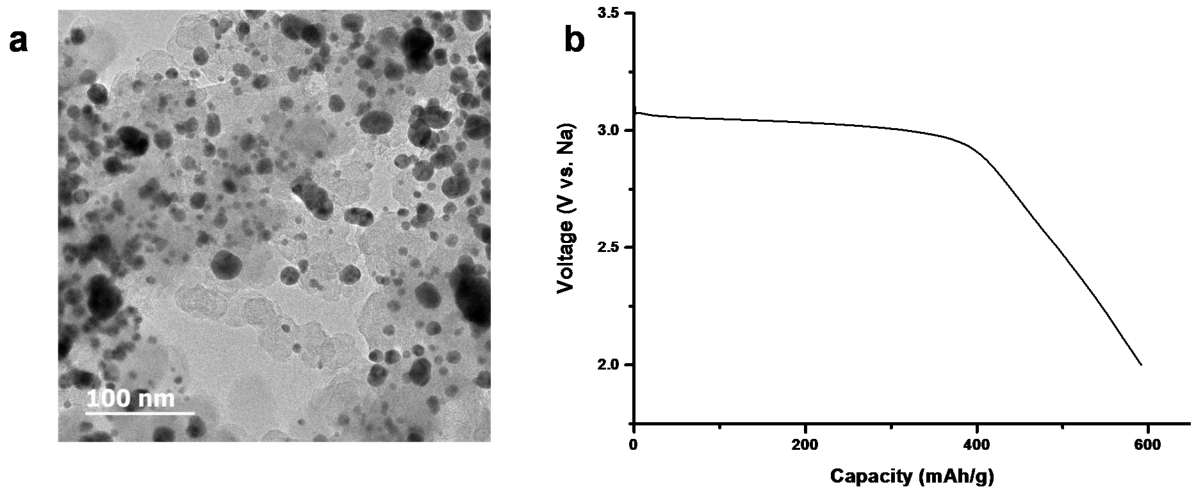

Figure S8. Characterization of Au@C cathode material used for SERS measurement. (a) A TEM image of the Au@C nanocomposite. (b) The discharge voltage profile of the Au@C cathode for the SERS measurement in Figure 3e.

Table S1. Calculated Raman frequency (cm^{-1}) and the assignment of vibration mode for each Raman frequency.

mode	ν(cm^{-1})	Raman Int.[a]	Vibration mode
1	420	4.9	O–Al–O bending
2	452	2.8	O–S–O bending
3	497	1.4	O–S–O bending
4	534	4.8	Cl–Al–Cl asymmetric stretching
5	564	0.8	O–Al–O asymmetric bending
6	631	1.8	O–Al–O symmetric stretching
7	807	0.6	S–O stretching
8	935	15.8	S–O stretching

[a] Raman intensities are given in units of $Å^4$ amu^{-1}

Table S2. Estimation of theoretical energy density

By, $2Na + NaAlCl_4 \cdot 2SO_2 \leftrightarrow 2NaCl + NaAlCl_2(SO_2)_2$,

	Discharge Capacity (mAh/g)	Specific Energy Density (Wh/kg)
Based on the mass of [catholyte]	168	503
Based on the mass of [discharge products]	147	440
Based on the mass of [catholyte + carbon]	153	460
Based on the mass of [discharge products + carbon]	136	407

1) Capacity based on the mass of carbon cathode: 1800 mAh/g (experimental data as shown Fig. 1)

2) Corresponding carbon mass for the reaction of 1g-catholyte: 0.0931 g

3) Operational voltage: 3 V

Scheme S1. Sequential description of the discharge reaction in a Na-SO_2 cell with a schematic illustration.

- At the Na-metal anode,

  $2Na \rightarrow 2Na^{+} + 2e^{-}$

- At the carbon cathode,

  [step 1] $2SO_2$ (in the complex) + $2e^{-} \rightarrow 2SO_2^{\bullet-}$

  [step 2] $2SO_2^{\bullet-} + Na^{+}\text{-}AlCl_4^{-} \rightarrow NaAlCl_2(SO_2)_2 + 2Cl^{-}$, where the $NaAlCl_2(SO_2)_2$ precipitates at the cathode

  [step 3] $2Cl^{-}$ (in the step 2) + $2Na^{+}$ (from the anode) $\rightarrow$ 2NaCl (also, precipitates at the cathode)

The above discharge reaction is schematically illustrated in the below:

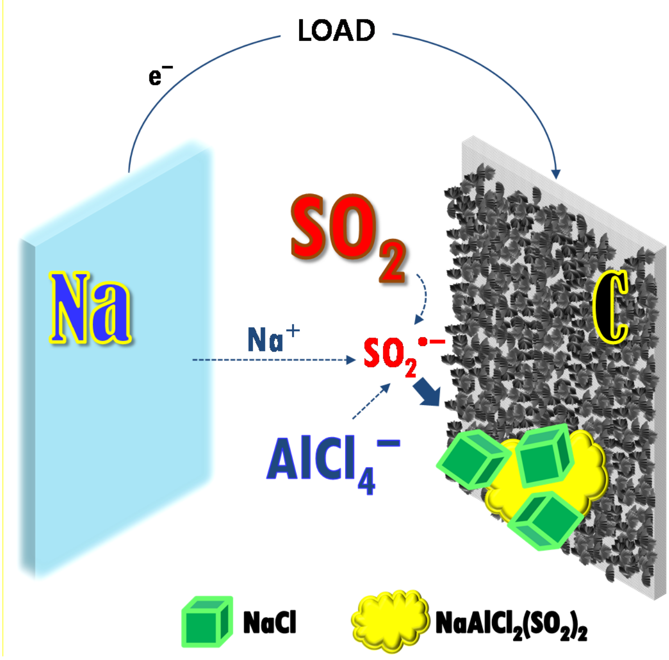

Supplement: Supplementary Information [file srep12827-s1.pdf]
